# Partial Response to Small Molecule Inhibition in a Case of Anaplastic Large Cell Lymphoma

**DOI:** 10.7759/cureus.23627

**Published:** 2022-03-29

**Authors:** Sarah Young, Amirah Kuzu, Mike Magill, Julio Hajdenberg

**Affiliations:** 1 Oncology, Orlando Regional Medical Center, Orlando, USA; 2 Pathology, Orlando Regional Medical Center, Orlando, USA

**Keywords:** mapk pathway, targeted therapy, binimetinib, histiocytic sarcoma, t-cell lymphoma

## Abstract

In the era of personalized medicine, small-molecule inhibitors have become key to targeting many malignancies. Multiple hematologic malignancies are driven by small-molecule pathways that are seemingly ripe for such targeting. In this case report, we present a patient who was treated with a mitogen-activated extracellular signal-regulated kinase (MEK) inhibitor for what was originally diagnosed as a histiocytic sarcoma. Re-biopsy ultimately revealed an anaplastic lymphoma kinase (ALK)-negative anaplastic large cell lymphoma (ALCL), but his disease initially showed a remarkable response to MEK inhibition. This case illustrates both the importance of obtaining high-quality biopsy specimens for diagnostic and molecular analysis as well as the need for further research into the molecular drivers of T-cell lymphomas that may be amenable to targeted therapies.

## Introduction

Small molecule inhibitors of the mitogen-activated extracellular signal-regulated kinase (MEK), a key component of the mitogen-activated protein kinase (MAPK) pathway, have emerged as important tools for the treatment of cancer, most notably melanoma. MEK inhibitors have also shown efficacy in histiocytic sarcoma, an aggressive and rare malignant neoplasm of mature histiocytes that is often difficult to diagnose due to overlapping features with other malignancies like anaplastic large cell lymphoma (ALCL) [1]. In fact, others have shown that a significant portion of malignancies initially diagnosed as "histiocytic" are later reclassified as other types of lymphoma [2]. Histiocytic sarcoma is driven by mutations in BRAF (V600E) and in the MAPK pathway [3]. A recent phase II study showed the efficacy of the MEK inhibitor cobimetinib in histiocytic neoplasms [4]. In this report, we present a patient who was treated with MEK inhibitors for what was originally diagnosed as a histiocytic sarcoma. Re-biopsy ultimately revealed an anaplastic lymphoma kinase (ALK)-negative ALCL, but his disease initially responded well to MEK inhibition.

## Case presentation

A 65-year-old Caucasian male presented to our institution to establish care for a recently diagnosed histiocytic neoplasm at a tertiary center. He first noticed a lump on his posterior scalp about eight months prior to the diagnosis of neoplasia at a local hospital. A wide local excision biopsy had shown increased eosinophils and a poorly differentiated neoplasm favoring histiocytic sarcoma. This neoplasm recurred one month after the initial resection.

Our patient was referred to an academic institution for further care. Restaging at that time with PET-CT imaging revealed innumerable bilateral pulmonary metastases as well as lymph node metastases in the neck. Pathology consultation regarding both of the initial specimens was read as a hematolymphoid neoplasm with histiocytic features and eosinophils. An additional bronchoscopy with the endobronchial ultrasound-guided aspiration of a PET-avid right lower lobe nodule revealed atypical histiocytic cells and abundant eosinophils. During subsequent staging, bone marrow biopsy exhibited limited involvement (<5%) by the neoplasm. A course of gemcitabine and taxotere was recommended for the treatment of histiocytic sarcoma, but the patient decided to return to Florida to continue with his care. Prior to his return, we were contacted by the academic center to participate in his treatment.

**Figure 1:**
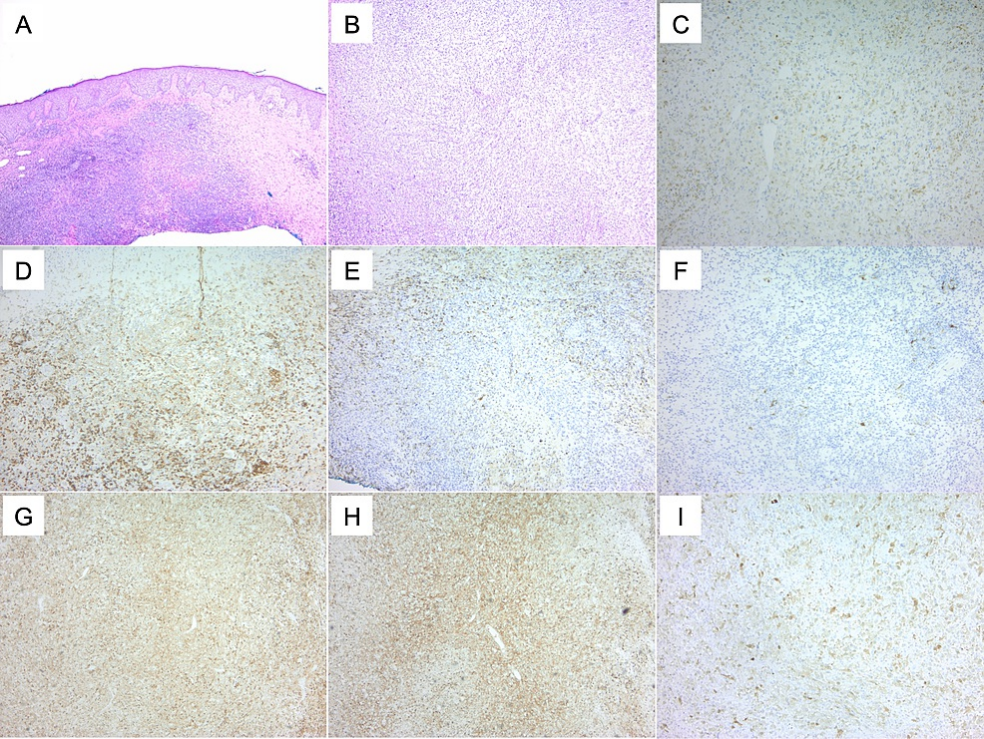
Histological features and biomarker expression in a case of T-cell lymphoma. [(A) H&E, (D) CD4, (E) CD45, (F) S100] compared to a case of histiocytic sarcoma [(B) H&E, (C) CD68, (G) CD4, (H) CD45, (I) S100]. While CD4 and CD45 can be expressed in both malignancies, positive staining for CD68 and S100 in histiocytic sarcoma aid in differentiation from the T-cell lymphoma. H&E: hematoxylin and eosin stain.

A larger biopsy of a skin tumor was recommended because we had some concerns about the small size and quality of the prior specimens and the ability to do a proper internal review. While this was obtained and processed for morphological and molecular profiling, we started treatment with the MEK inhibitor binimetinib for a presumed diagnosis of histiocytic sarcoma. Pathology from the new specimen ultimately revealed a T-cell lymphoma (Figure 1), expressing CD30 with rearranged gamma chains of the T-cell receptor. Our final diagnosis was stage IV ALK-negative anaplastic large cell lymphoma. Additional testing was negative for DUSP22 rearrangement and positive for a mutation in KRAS (G12S).

**Figure 2:**
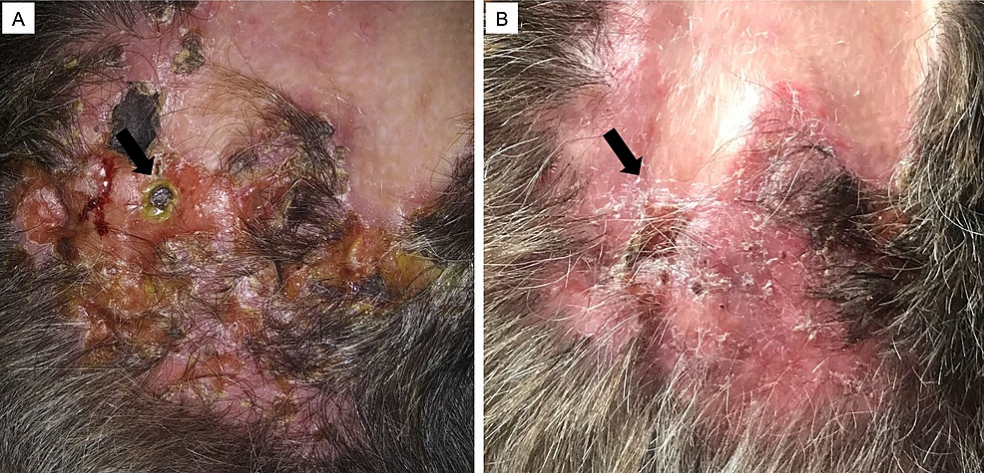
Clinical images of scalp lesions (arrow) before (A) and after (B) treatment with a MEK inhibitor. MEK: mitogen-activated extracellular signal-regulated kinase.

**Figure 3:**
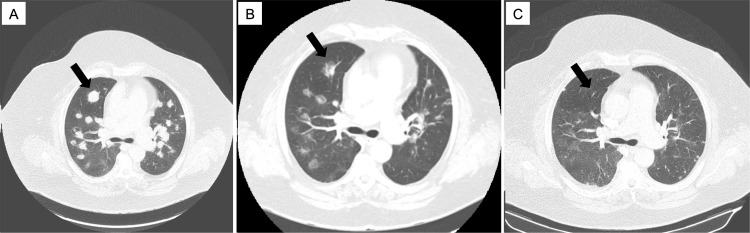
Computed tomography scans showing lung metastatic disease (arrow) at presentation. MEK: mitogen-activated extracellular signal-regulated kinase.

In the few weeks between starting binimetinib and confirmation of this new diagnosis, the patient experienced a substantial clinical improvement in his scalp lesions (Figure 2). Restaging CTs after five weeks of treatment with the MEK inhibitor revealed a partial response in the scalp, neck, and lung disease (Figure 3). Given the significant response, he was continued on the MEK inhibitor for a total of 12 weeks prior to the transition to standard first-line treatment for ALCL with brentuximab vedotin plus cyclophosphamide, doxorubicin, and prednisone (BV+CHP).

## Discussion

The MAPK cascade, including the downstream Ras-ERK pathway, is well known for its contribution to tumorigenesis and is thought to be altered in up to a third of all cancers. MEK inhibitors, including binimetinib, block a key component of this pathway. These allosteric kinase inhibitors bind to a pocket near the catalytic site and induce a conformation change that reduces kinase activity, allowing for increased specificity and decreased off-target effects [5]. While MEK inhibitors have been investigated in many other tumor types, including histiocytic sarcoma as described above, there is little information on their use in T-cell lymphoma.

Anaplastic large cell lymphoma is a CD30-positive T-cell lymphoma that can be divided into two distinct subtypes, ALK-positive and ALK-negative, based on rearrangement of the ALK gene [6,7]. ALK-negative ALCL continues to have a worse prognosis compared to ALK-positive ALCL, with a five-year overall survival rate of <50% [8]. The recent addition of brentuximab vedotin to front-line multi-agent chemotherapy has shown an impressive improvement in outcomes [9]. Additionally, the investigation into a novel, targeted therapies for ALCL continues, with the approval of dihydrofolate (DHFR) and histone deacetylase (HDAC) inhibitors in the relapsed/refractory setting. Interestingly, recent studies have revealed multiple molecular subtypes and abnormally regulated pathways that may contribute to ALK-negative ALCL tumorigenesis [10], including DUSP22 and TP63 rearrangements [11], aberrant ERBB4 expression [12], and activation of the JAK-STAT3 pathway [13]. Each of these offers a new avenue of investigation into molecularly targeted therapeutics specific to ALK-negative ALCL.

Our patient was initiated on a MEK inhibitor due to a mistaken diagnosis of histiocytic sarcoma based on a suboptimal biopsy specimen. Hematologic malignancies can be very challenging to diagnose, as evidenced by discordance among pathologists and later reclassification of rare entities such as T-cell lymphomas [14] and histiocytic neoplasms [2]. Therefore, high-quality biopsy specimens are essential for appropriate morphologic, immunohistochemical, and molecular evaluation of such malignancies, as highlighted by this case.

Fortunately, in the few weeks before he was ultimately diagnosed with ALK-negative ALCL based on a superior biopsy specimen, our patient had a clinically significant response to the MEK inhibitor binimetinib. Further testing revealed that our patient’s neoplasm harbored an activating mutation in KRAS (G12S), an upstream activator of the Ras-ERK pathway, which may partially explain the activity of MEK inhibition in this case. Of note, MEK inhibitor responses in other cancers with activating KRAS mutations have been somewhat disappointing, possibly due to a cytostatic rather than cytotoxic effect of MEK inhibition following upstream mutations [5,15]. As such, other mechanisms explaining the effect of MEK inhibition may account for the response seen in our case. For example, CD30, which is highly expressed in ALCL, is known to signal through the MAPK pathway [16,17]. If CD30 signaling contributes to the propagation of hematologic malignancies as suggested in previous studies [18], then we can hypothesize that MEK inhibition would also be a viable treatment option in ALK-negative ALCL.

## Conclusions

When dealing with rare hematologic neoplasms, every effort should be made to secure generous amounts of tissue so that extensive immunohistochemical and molecular testing can be performed. To the best of our knowledge, the KRAS G12S mutation reported here has not been previously reported in this type of T-cell neoplasm. In addition, in this particular case of non-DUSP22 rearranged, ALK-negative ALCL, it gave us the opportunity to evaluate an individualized treatment.

ALK-negative ALCL is a genetically heterogeneous disease that is suitable for the investigation of new targeted therapies. In our patient’s case, the success of MEK inhibition suggests that signaling through the MAPK pathway was critical to lymphomagenesis. We suggest that MEK inhibitors represent an area of interest in the targeted treatment of ALK-negative ALCL in the future.

## References

[1] Pan Z, Xu ML (2019). Histiocytic and dendritic cell neoplasms. Surg Pathol Clin.

[2] Vos JA, Abbondanzo SL, Barekman CL, Andriko JW, Miettinen M, Aguilera NS (2005). Histiocytic sarcoma: a study of five cases including the histiocyte marker CD163. Mod Pathol.

[3] Diamond EL, Durham BH, Haroche J (2016). Diverse and targetable kinase alterations drive histiocytic neoplasms. Cancer Discov.

[4] Diamond EL, Durham BH, Ulaner GA (2019). Efficacy of MEK inhibition in patients with histiocytic neoplasms. Nature.

[5] Yaeger R, Corcoran RB (2019). Targeting alterations in the RAF-MEK pathway. Cancer Discov.

[6] Hapgood G, Savage KJ (2015). The biology and management of systemic anaplastic large cell lymphoma. Blood.

[7] Shustov A, Soma L (2019). Anaplastic large cell lymphoma: contemporary concepts and optimal management. Cancer Treat Res.

[8] Savage KJ, Harris NL, Vose JM (2008). ALK- anaplastic large-cell lymphoma is clinically and immunophenotypically different from both ALK+ ALCL and peripheral T-cell lymphoma, not otherwise specified: report from the International Peripheral T-Cell Lymphoma Project. Blood.

[9] Horwitz S, O'Connor OA, Pro B (2019). Brentuximab vedotin with chemotherapy for CD30-positive peripheral T-cell lymphoma (ECHELON-2): a global, double-blind, randomised, phase 3 trial. Lancet.

[10] Mereu E, Pellegrino E, Scarfò I, Inghirami G, Piva R (2017). The heterogeneous landscape of ALK negative ALCL. Oncotarget.

[11] Parrilla Castellar ER, Jaffe ES, Said JW (2014). ALK-negative anaplastic large cell lymphoma is a genetically heterogeneous disease with widely disparate clinical outcomes. Blood.

[12] Scarfò I, Pellegrino E, Mereu E (2016). Identification of a new subclass of ALK-negative ALCL expressing aberrant levels of ERBB4 transcripts. Blood.

[13] Crescenzo R, Abate F, Lasorsa E (2015). Convergent mutations and kinase fusions lead to oncogenic STAT3 activation in anaplastic large cell lymphoma. Cancer Cell.

[14] Chan JK, Kwong YL. (2010). Common misdiagnoses in lymphomas and avoidance strategies. Lancet Oncol.

[15] Neuzillet C, Tijeras-Raballand A, de Mestier L, Cros J, Faivre S, Raymond E (2014). MEK in cancer and cancer therapy. Pharmacol Ther.

[16] Zheng B, Fiumara P, Li YV (2003). MEK/ERK pathway is aberrantly active in Hodgkin disease: a signaling pathway shared by CD30, CD40, and RANK that regulates cell proliferation and survival. Blood.

[17] van der Weyden CA, Pileri SA, Feldman AL, Whisstock J, Prince HM (2017). Understanding CD30 biology and therapeutic targeting: a historical perspective providing insight into future directions. Blood Cancer J.

[18] Watanabe M, Sasaki M, Itoh K (2005). JunB induced by constitutive CD30-extracellular signal-regulated kinase 1/2 mitogen-activated protein kinase signaling activates the CD30 promoter in anaplastic large cell lymphoma and reed-sternberg cells of Hodgkin lymphoma. Cancer Res.

